# Ultrasound-Treated Sweet Potato Peel Enhances Nutritional Quality and Antioxidant Potential of Gluten-Free Brownies: A Metabolomics and Cell-Based Approach

**DOI:** 10.3390/antiox15060753

**Published:** 2026-06-15

**Authors:** Pablo Ayuso, Rocío Peñalver, Jhazmin Quizhpe, Pascual García-Pérez, Gema Nieto

**Affiliations:** Department of Food Technology, Nutrition and Food Science, Veterinary Faculty, Campus de Excelencia Internacional de Ámbito Regional (CEIR), Campus Mare Nostrum (CMN), University of Murcia, 30100 Murcia, Spain; pablo.ayuson@um.es (P.A.); rocio.penalver@um.es (R.P.); pascual.garcia@um.es (P.G.-P.)

**Keywords:** foodomics, gluten-free, bioactive compounds, mixomics, anti-inflammatory

## Abstract

Agri-food by-products such as sweet potato peel (SP) represent a sustainable and valuable source of bioactive compounds for improving gluten-free (GF) foods. This study evaluated the nutritional and functional impact of incorporating SP at 8% and 16%, either untreated or ultrasound-assisted extraction (UAE)-treated, into GF brownies. An untargeted metabolomics approach combined with chemometrics was applied to characterize phytochemical modulation after in vitro digestion of the brownies, while antioxidant and anti-inflammatory effects were assessed using RAW264.7 macrophages. SP incorporation increased the dietary fiber (reaching a content of 7.86%) and glycosylated flavonoid content in reformulated brownies, leading to a reduction of inflammatory markers in the cellular model. Sensory evaluation showed that SP addition did not significantly affect texture-related attributes or extract-related perception. In contrast, UAE acted as an efficient extraction strategy, enhancing terpenoid-like compounds and total phenolic content (TPC), reaching values of 401.97 mg GAE 100 g^−1^ after 16% incorporation. Overall, combining SP valorization with UAE represents a promising strategy to develop nutritionally enhanced GF products, providing a foodomics-based framework for next-generation functional bakery products.

## 1. Introduction

The gluten-free (GF) market has experienced substantial growth, with an estimated annual growth rate of 7.6% between 2020 and 2027 [1]. This expansion is largely driven by the rising prevalence of celiac disease [2]. However, current trends also indicate a growing interest among consumers without gluten-related disorders, who are increasingly attracted to GF products due to their perceived health benefits [3]. According to the European Commission, gluten-free products are defined as foods containing no more than 20 mg/kg (20 ppm) of gluten [4], which may include products naturally free of gluten-containing cereals or foods specifically formulated, processed, or manufactured to reduce their gluten content to below this threshold. Recent studies highlight that many commercially available GF bakery products, typically formulated with refined starch-based flours such as rice or corn, exhibit imbalanced nutritional profiles compared to their wheat-based counterparts, often characterized by lower levels of dietary fiber, iron, and calcium, as well as reduced amounts of bioactive compounds [5]. In addition, comparative studies have shown that gluten-free bakery products generally present lower protein quality, dietary fiber content, and micronutrient density than conventional wheat-based baked goods, mainly due to the absence of gluten-containing cereals and the frequent use of refined starches as substitutes [6,7]. Consequently, significant research efforts have been directed toward improving the nutritional quality of GF foods. In this context, innovative formulations and processing techniques have been developed for products such as muffins [8], sourdough bread [9], and cookies [10] with the aim of enhancing their nutritional value and providing healthier alternatives for consumers.

Sweet potato peels (*Ipomoea batatas* L.) have gained increasing attention as a valuable source of bioactive compounds with promising applications in the food industry. These by-products refer to the outer periderm and cortex layers of the tuber that are removed during industrial or laboratory peeling processes. Peels are typically generated through mechanical methods such as knife peeling, abrasive peeling, or steam peeling [11], which may result in variable amounts of adhering flesh depending on the processing conditions and efficiency. Consequently, “peel” fractions can contain minor residual pulp, and their composition may therefore vary accordingly. These by-products exhibit notable antioxidant, anti-inflammatory, and anticancer properties [12], largely attributed to their richness in polyphenols such as flavonols (quercetin and kaempferol derivatives), phenolic acids (mainly chlorogenic acid) and lignans (pinoresinol and matairesinol) [13]. Additionally, certain varieties, particularly purple-fleshed sweet potatoes, are especially rich in flavonols (i.e., kaempferol and quercetin derivatives) and anthocyanins (i.e., peonidin and delphinidin derivatives), which further enhance their antioxidant capacity and functional value [14]. In parallel, the increasing generation of agri-food by-products has raised significant environmental and economic concerns, driving interest in their valorization as functional ingredients capable of improving both the technological and nutritional properties of food products. These materials, which are usually discarded as waste, present a commercial opportunity for their revaluation due to their high fiber and bioactive compound content. According to the FAO, up to 45% of tubers are lost during the entire postharvest chain, including storage, handling, and industrial processing; in the case of sweet potatoes, this represents a significant volume of potentially valorizable material [15].

In this context, the application of emerging green technologies has been proposed as a sustainable strategy to efficiently recover valuable compounds from plant by-products. Ultrasound-assisted extraction has been claimed to be an extraction technique capable of effectively recovering compounds of interest in plant by-products. This technique uses ultrasonic waves (>20 kHz) to cause cavitation bubbles to collapse, producing mechanical effects such as fragmentation, pore formation, and increased cell wall permeability, thereby facilitating the release of bioactive compounds into the solvent [16]. UAE has demonstrated high efficiency in extracting phenolic compounds from citrus peels, apple pomace, and flax seeds [17] as well as natural pigments, including carotenoids, anthocyanins, and betalains, from fruit and vegetable by-products [18]. Moreover, this technique has also proven effective for the recovery of pectins from grape pomace, jackfruit peel, and pomegranate peel [19]. In comparison with conventional extraction methods (such as maceration or solid–liquid extraction), UAE generally provides higher extraction yields, shorter processing times, and reduced solvent consumption due to enhanced mass transfer mechanisms driven by cavitation phenomena [16,20]. However, conventional methods remain widely used because of their simplicity and low equipment cost, although they are typically less efficient and more time-consuming than UAE-based approaches [20].

Recent research has highlighted the potential of metabolomics and chemometric approaches as powerful tools for the comprehensive characterization of complex biological and food matrices. Unlike targeted metabolomics, which focuses on predefined metabolites, untargeted metabolomics enables the comprehensive and unbiased profiling of a broad spectrum of compounds, facilitating the discovery of unexpected metabolic changes and novel biomarkers in complex food systems [21]. The use of advanced bioinformatic analyses and multivariate techniques (i.e., principal component analysis (PCA), hierarchical cluster analysis (HCA), and orthogonal partial least squares discriminant analysis (OPLS-DA) and regularized canonical correlation analysis (rCCA), enables the interpretation of large and complex MS/MS datasets. These approaches facilitate the detection of biologically meaningful changes, offering a holistic perspective on how processing, storage, and reformulation affect the chemical profile of foods. Of particular interest is the application of untargeted metabolomics to explore the fate of bioactive compounds throughout gastrointestinal digestion, enabling the identification and semi-quantification of phytochemicals released or transformed during digestion and thus providing a comprehensive picture of their bioaccessibility [22]. This is especially relevant in the development of functional foods [23,24,25], where reformulations often involve the incorporation of bioactive compounds, plant extracts, or novel ingredients aimed at improving nutritional and health-promoting properties.

In this study, untargeted metabolomics combined with chemometrics was applied to characterize the large-scale chemical impact of UAE treatment on sweet potato peel by-products, as well as the metabolomic modulation occurring after their incorporation into gluten-free brownies, both before and after in vitro gastrointestinal digestion. In parallel, the potential of these by-products as functional ingredients was evaluated through nutritional analysis, assessment of antioxidant capacity and total phenolic content, and a cell-based anti-inflammatory assay using RAW264.7 macrophages. This integrative approach aims to advance the valorization of agri-food by-products while providing a foodomics-based framework for the development of nutritionally enhanced gluten-free products.

## 2. Materials and Methods

### 2.1. Processing of Sweet Potato By-Products

The purple-skinned sweet potatoes (*Ipomoea batatas*), obtained from a local supermarket, were washed and manually peeled, separating the by-product from the edible portion. Peels were then dehydrated in a forced-air oven at 50 °C for 24 h. The resulting dry material was ground and sieved to obtain a fine flour with a particle size of 300 µm, following the procedure described by Ayuso et al. (2024) [26]. The dried powder was subjected to ultrasound-assisted extraction (UAE) using a Hielscher UIP500hdT ultrasound processor (Hielscher Ultrasonic GmbH, Teltow, Germany). Sweet potato peel (SP) flour was mixed with a solution of ethanol and water in a 60:40 ratio (*v*/*v*), at a solid/liquid ratio of 1:20 (*w*/*v*). Extraction conditions of 20 kHz frequency and 210 W power were employed, with a total time of 6 min. Temperature was monitored throughout the process using a built-in thermocouple. No external cooling system was applied; however, due to the short extraction time (6 min) and moderate ultrasound power settings, the maximum temperature did not exceed 26.5 °C. After extraction, the suspension was filtered through a 100 µm mesh to remove the solid phase. Ethanol was removed using a Hei-VAP Core rotary evaporator (Heidolph Instruments, Schwabach, Germany) and the obtained aqueous solution was subsequently freeze-dried (LE-LYOEPIC85 freeze dryer, Dara-Lyo, Barcelona, Spain) and sieved to obtain a fine flour of 300 µm. The dried powder derived from the UAE treatment was designated as SPU (sweet potato peel treated with UAE).

### 2.2. Preparation of Gluten-Free Brownies

**Figure 1:**
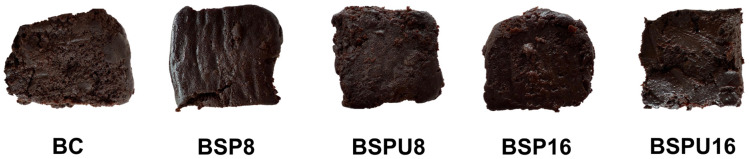
Visual appearance of GF brownies. Abbreviations: BC: control brownie; BSP8: brownie with 8% SP; BSPU8: brownie with 8% SPU; BSP16: brownie with 16% SP; BSPU16: brownie with 16% SPU.

Buckwheat flour, sugar, whole milk, extra virgin olive oil (EVOO), 70% cocoa powder, chocolate chips, and eggs were purchased at a local supermarket (Murcia, Spain). These ingredients, including the sweet potato by-products, were mixed and whipped in a bowl for 2 min, following the quantities described in Table 1. The dough was baked at 180 °C for approximately 20 min using silicone moulds (24 × 24 cm) in a convection and steam oven. After baking, brownies were allowed to cool at room temperature for 1 h. Five different GF brownie formulations were prepared in separate batches: control brownie (BC); brownie with 8% sweet potato peel (BSP8); brownie with 8% sweet potato peel treated with UAE (BSPU8); brownie with 16% sweet potato peel (BSP16); and brownie with 16% sweet potato peel treated with UAE (BSPU16). The percentage of sweet potato by-product incorporation was calculated in relation to the amount of flour used in the formulation. The visual appearance of the GF brownies is shown in Figure 1. Samples destined for nutritional, physicochemical and antioxidant analysis were finely ground and stored at −20 °C. Finally, the GF brownies destined for the metabolomic analysis were freeze-dried using an LE-LYOEPIC85 freeze dryer (Dara-Lyo, Barcelona, Spain).

### 2.3. Proximate Composition

The proximate composition of GF brownies and SP by-products was determined following the procedures of the Association of Official Analytical Chemists [27]. The moisture (964.22), protein (955.04), fat (920.39), ash (923.03), soluble, insoluble, and total dietary fiber (991.43) contents were determined. Energy and carbohydrate content were calculated following the guidelines established by the Food and Agriculture Organization of the United Nations (FAO) [28]. All nutritional analyses were performed in triplicate.

### 2.4. Color, pH, and Total Titratable Acidity (TTA)

Color measurements of GF brownies were performed according to the CIEL*a*b* system. Lightness (L*), redness (a*), yellowness (b*), chroma (C), and hue (h) parameters were recorded using a Konica Minolta CR-400 colorimeter (Minolta, Tokyo, Japan). The total color differences (ΔE*) were determined following the equation, where s and c indicate the sample and control measurements, respectively:

(1) $$\Delta E^{*}=\sqrt{{\left(L_{s}^{*}-L_{c}^{*}\right)}^{2}+{\left(a_{s}^{*}-a_{c}^{*}\right)}^{2}+{\left(b_{s}^{*}-b_{c}^{*}\right)}^{2}}$$

The pH values of brownies were measured using a sensION+ PH31 pH meter (Hach-Lange, Barcelona, Spain), following ISO guidelines. Determination was performed after mixing the sample with distilled water in a 1:10 proportion (*w*/*v*).

Total titratable acidity (TTA) of GF brownies was determined according to AACC method 02–31.01 [29] and expressed as the volume (mL) of 0.1 M NaOH required for neutralization. Color, pH and TTA measurements were performed in triplicate.

### 2.5. In Vitro Gastrointestinal Digestion

GF brownies were subjected to an in vitro digestion applying the INFOGEST 2.0 procedure, described by Brodkorb et al. (2019) [30]. This process consisted of three sequential phases: oral, gastric, and intestinal. For the oral phase, two grams of brownie were mixed with 3 mL of distilled water and 5 mL of simulated salivary fluid (SSF), containing human salivary α-amylase (75 U mL^−1^). The oral phase was simulated by adjusting the pH to 7.0 and incubating the samples for 2 min at 37 °C with constant agitation. Subsequently, the resulting mixture was mixed in a 1:1 (*v*/*v*) ratio with simulated gastric fluid (SGF) containing porcine pepsin 2000 U mL^−1^ to complete the gastric phase. The pH was acidified to 3.0, and digestion was carried out for 120 min at 37 °C under constant stirring. Then, the gastric chyme was mixed with simulated intestinal fluid (SIF), supplemented with porcine pancreatin based on trypsin activity (100 U mL^−1^) and bile salts (10 mM). The pH was neutralized to 7.0 and the final digests were incubated for 120 min at 37 °C. Finally, the resulting mixture was centrifuged at 9000× *g* for 10 min (FC5718R centrifuge, OHAUS Corp., Parsippany, NJ, USA) to separate the digested fraction from the solid residues. Each digestion was performed in triplicate. An individual aliquot was collected for the determination of antioxidant activity, total phenolic content (TPC), and subsequent cell culture assays. The resulting digests were freeze-dried prior to metabolomics analysis using an LE-LYOEPIC85 freeze dryer (Dara-Lyo, Barcelona, Spain).

### 2.6. Total Phenolic Content (TPC) and Antioxidant Activity Analysis

Two grams of GF brownies and SP by-products were homogenized in 8 mL of a methanol/water mixture (80:20, *v*/*v*). The mixture was kept at 4 °C for 24 h until complete extraction, centrifuged at 4500 rpm for 25 min (FC5718R centrifuge, OHAUS Corp., Parsippany, NJ, USA), and filtered through 0.45 µm membranes. The in vitro digestions of GF brownies were directly analyzed. Antioxidant activity was determined through, 2,2-diphenyl-1-picrylhydrazyl (DPPH) radical scavenging activity, radical cation scavenging capacity of 2,2-azinobis (3-ethylbenzothiazolin)-6-sulphonic acid (ABTS), and ferric reducing antioxidant power (FRAP) analysis. The DPPH assay was performed according to Brand-Williams et al. (1995) [31]. A total of 100 µL of the extracted brownie, sweet potato by-product or digested sample was mixed with 3.9 mL of a 0.064 mM DPPH solution. The mixture was kept for 30 min in the dark, absorbance was read at 515 nm. The ABTS assay was performed following the method described by Re et al. (1999) [32]. A total of 100 µL of the extracted sample was mixed with 1 mL of ABTS working solution, and absorbance was measured at 734 nm after 2 min of incubation. Working solution consisted of a 7 mM ABTS solution prepared with 2.45 mM potassium persulfate and incubated in the dark for 16 h. FRAP analysis was performed following Benzie & Strain (1996) [33]. A total of 1 mL of FRAP reagent was mixed with 100 µL of the extracted sample and absorbance was measured at 593 nm after 4 min of incubation. The FRAP reagent was prepared by mixing 300 mM acetate buffer, 10 mM 2,4,6-tripyridyl-s-triazine (TPTZ) solution, and 20 mM FeCl_3_·6H_2_O solution in a 10:1:1 (*v*/*v*/*v*) ratio. Antioxidant activity (ABTS, DPPH and FRAP) was quantified using a trolox standard curve prepared in methanol. Results were expressed as µM Trolox equivalents (TE) per 100 g^−1^ of fresh weight (FW) sample.

Total phenolic content (TPC) was assessed with the Folin–Ciocalteu method (Singleton et al., 1999) [34]. A total of 100 µL of the extracted sample was mixed with 500 µL of Folin–Ciocalteu reagent and 400 µL of a Na_2_CO_3_ solution (7.5% (*w*/*v*)). TPC results were expressed as mg of gallic acid equivalents (GAE) per 100 g^−1^ of FW sample, using a gallic acid standard curve prepared in methanol. TPC and antioxidant assays were performed with three independent replicates using a UV21 spectrophotometer (ONDA, Carpi, Italy).

### 2.7. Untargeted Metabolomic Analysis Through UHPLC-QTOF-HRMS

The sweet potato by-products and GF brownies were subjected to an untargeted metabolomic analysis. Prior to analysis, 0.5 g of lyophilized SP by-products and brownies were mixed with 5 mL of an 80% (*v*/*v*) methanol solution acidified with 0.1% formic acid. For the brownie in vitro digests, lyophilized powder was mixed with 12 mL of a 50% (*v*/*v*) methanol solution with 0.1% formic acid. All samples were then centrifuged at 8000× *g* for 10 min at 4 °C using an FC5718R centrifuge (OHAUS Corp., Parsippany, NJ, USA). The resulting supernatants were filtered through 0.22 μm membrane filters.

Untargeted metabolomic analysis was performed through ultra-high-performance liquid chromatography coupled to quadrupole-time-of-flight high-resolution mass spectrometry (UHPLC-QTOF-HRMS). Chromatographic separation was performed on a 1290 Infinity II UHPLC system (Agilent Technologies^®^, Santa Clara, CA, USA) equipped with a Poroshell 120 PFP reversed-phase column (2.1 × 100 mm, 1.9 µm particle size; Agilent Technologies^®^). The mobile phase comprised water (solvent A) and acetonitrile (solvent B), both containing 0.1% (*v*/*v*) formic acid. A gradient elution program was applied, reducing solvent A from 94% to 6% over 32 min at a flow rate of 0.2 mL min^−1^, with an injection volume of 6 µL. Mass spectrometric analysis was conducted using a G6550 QTOF mass spectrometer equipped with a JetStream electrospray ionization (ESI) source (Agilent Technologies^®^, Santa Clara, CA, USA). High-resolution data acquisition was conducted in positive ionization mode (ESI+) across a full scan range of 100–1200 *m*/*z* at 1 spectrum s^−1^, providing a resolution of 30,000 FWHM. The nebulizer operated at 310.3 kPa, while the nozzle and capillary voltages were set to 3500 V and 4000 V, respectively. Nitrogen was used as sheath gas (12 L min^−1^ at 315 °C) and drying gas (14 L min^−1^ at 250 °C). Samples were acquired in MS-only and MS2 modality. Brownie extracts and SP by-products were analyzed using six independent replicates. In vitro digests were injected in duplicate, resulting in six replicates per treatment (*n* = 6).

The identification and annotation of chemical features was performed using MS-DIAL software (v5.5.250820). Alignment was achieved by automatic peak detection after LOWESS normalization, and compound annotation was obtained by spectral comparison with three publicly available MS/MS databases: FooDB, BMDMS-NP, and Fiehn/Vaniya natural product library [35]. An accurate mass tolerance of 0.05 Da was established for MS1 and 0.1 Da for MS2. A retention time tolerance for alignment of 0.1 min and an MS1 tolerance for alignment of 0.025 Da were set. Minimal peak height was set at 10,000 cps. The ESI+ adducts [M + H]^+^, [M + CH_3_OH + H]^+^, [M + H_2_O]^+^, [M + ACN]^+^, and [M + Na]^+^ were excluded based on the analytical method. The identification score cut-off was set at 67%, and a peak count filter of 8% was applied, retaining only metabolite features detected in a minimum of 8% of the total sample set, to reduce noise and exclude low-prevalence features from downstream analysis. Metabolite annotation was performed in accordance with the Level 2 of the COSMOS Metabolomics Standards Initiative (putatively annotated compounds [36]. In addition, the identified phenolic compounds present in SP by-products and raw and digested brownies were subjected to a semi-quantitative analysis, grouping compounds from the same family and quantifying them relative to a representative standard for each family. Luteolin was used as a reference standard for flavonoids (y = 318,072x; R^2^ = 0.989), and chlorogenic acid for phenolic acids (y = 11,791x; R^2^ = 0.999). Sesamin was used as a representative standard for low-molecular-weight (LMW) phenolics and other polyphenols (y = 906.34x; R^2^ = 0.980) for semi-quantitative purposes, without implying structural representativeness of all compounds within this group. Semi-quantitative results were expressed as mg of each reference standard equivalent per 100 g of DW product. The percentage of post-digestion bioaccessibility (BA) was obtained following the formula, where C_dig_ stands for the content of digested samples, and C_raw_ stands for the content of raw samples:

(2) BA % = (C_dig_/C_raw_) × 100

### 2.8. Inflammation Assay in RAW264.7 Macrophages

RAW264.7 macrophages (n°86010202) were supplied by the European Cell Culture Collection (ECACC, Salisbury, UK). The cell culture model was established following the methodology described by Serrano et al. (2019) [37] with small modifications. RAW264.7 macrophages were plated at a density of 1 × 10^4^ cells per well in 96-well culture plates and maintained in Dulbecco’s Modified Eagle Medium (DMEM; 4.5 g L^−1^ glucose, 2% glutamine, 2% streptomycin, 10% fetal bovine serum) under standard conditions (37 °C, 7.5% CO_2_), being the medium renewed every 2 days. GF brownies previously subjected to in vitro gastrointestinal digestion were filtered through 0.22 μm membranes and then mixed with DMEM in a 3% (*v*/*v*) proportion. This mixture was then incorporated to RAW264.7 macrophages for 24 h. Cell viability after brownie digests incorporation was evaluated using 3-(4,5-dimethylthiazol-2-yl)-2,5-diphenyltetrazolium bromide assay (MTT) [38]. After incubation, the enriched medium was removed and lipopolysaccharide (LPS) from *E. coli* (1 μg mL^−1^) mixed with standard medium was added to induce an inflammatory response. Negative and positive controls were included, consisting in the digestion blank applied to the cell culture model either without LPS stimulation (negative control) or with LPS treatment (positive control). After 24 h incubation, supernatants were collected to determine nitric oxide (NO) and inflammatory cytokines. RAW264.7 cells were harvested to assess intracellular reactive oxygen species (ROS) production. The experiments were conducted with RAW264.7 macrophages in passage 7, with six independent replicates per brownie formulation. This experiment was approved by the Biosafety Committee for Experimental Research of the University of Murcia, with code 717/2025.

Intracellular ROS after induction of inflammation were measured following the protocol described by Wan et al. (2015) [39]. Cells were preloaded with 12.5 µM 2′-7′-dichlorofluorescein diacetate (DCFH-DA) mixed with PBS and then incubated for 30 min at 37 °C. Fluorescence measurements were performed by a microplate reader (FLUOstar Omega, BMG Labtech, Ortenberg, Germany), using the MARS Data Analysis Software version 3.10 (BMG Labtech, Ortenberg, Germany) at λ_ex_ = 485 and λ_em_ = 530. The results were expressed as a percentage of ROS production in relation to the positive control treated with LPS.

Nitric oxide (NO) levels after LPS induction were quantified using a NO colorimetric assay kit (Thermo Scientific, Rockford, IL, USA). Data were presented as millimolar (mM) concentrations of NO in the cell culture supernatant.

IL-1β and IL-6 and tumour necrosis factor alpha (TNF-α) proinflammatory cytokines were determined using a Luminex MAGPIX^®^ system (Luminex Corporation, Austin, TX, USA). Cell supernatants were analyzed using a MILLIPLEX^®^ MAP Mouse Cytokine/Chemokine Magnetic Bead Panel (MCYTOMAG-70K; Merck Millipore, Billerica, MA, USA) following the instructions of the manufacturer. The results were expressed as pg mL^−1^ of each individual cytokine in the cell culture supernatant.

### 2.9. Sensory Analysis

The organoleptic characteristics of the GF brownie formulations were evaluated through a quantitative descriptive sensory analysis with eleven trained panelists (aged 21–46) following International Organization for Standardization (ISO) guidelines [40]. The study protocol was approved by the Bioethics Committee of the University of Murcia (M10/2024/444). Two training sessions focused on the development of sensory descriptors related to odor, flavor and texture parameters, and sweet potato peel recognition were conducted. Sensory analyses were carried out in individual booths under controlled environmental conditions. Each sample (approximately 20 g) was served in coded and randomly randomized white plastic plates. Panelists were instructed to cleanse their palate with still water between samples. The evaluation was conducted in a single session lasting approximately 45 min. Panelists assessed color, odor, and flavor using a 5-point intensity scale, incorporating descriptors linked to the added SP by-products, where 1 represented very low intensity (or absence of the attribute), 3 represented moderate intensity, and 5 represented very high intensity of the evaluated attribute. Texture attributes, including hardness, adhesiveness, sponginess, gumminess, chewiness, and cohesiveness perception, were also evaluated.

### 2.10. Statistical and Chemometrics Analysis

Statistical differences between GF brownie formulations were evaluated by one-way analysis of variance (ANOVA) followed by Tukey’s honestly significant difference (HSD) post hoc test. Differences between sweet potato by-products were assessed using Student’s *t*-test. Statistical significance was set at *p* < 0.05 and data was presented as mean ± standard deviation (SD). All statistical analyses were performed using version 30.0 of SPSS Statistics (IBM Corp., Armonk, NY, USA). Finally, the sensory results were normalized before statistical evaluation and a PCA biplot was subsequently generated in R using the factoextra package.

Multivariate analysis of the untargeted metabolomic dataset of SP by-products and raw and digested GF brownies was conducted using the online platform MetaboAnalyst 6.0 (https://www.metaboanalyst.ca, accessed on 12 February 2026). Raw data was filtered using a threshold of 40% of the interquartile range, and abundances were then median-normalized, log_2_-transformed, and auto-scaled prior to analysis. Hierarchical cluster analysis (HCA) was performed separately for SP by-products and raw and digested brownies using Euclidean distances and Ward’s method, based on normalized fold-change values and visualized through an integrative heatmap. In parallel, a principal component analysis (PCA) was conducted to explore the overall variations in the untargeted analysis. The proportion of variance explained by the data (R^2^) was determined, and statistical significance was assessed by multivariate permutation analysis of variance (PERMANOVA; *α* = 0.05). In addition, a Volcano analysis was carried out to identify the differentially accumulated metabolites (DAMs) in order to explore the changes between UAE treatment of by-products and brownie formulations. A fold-change threshold was set at |±2|, and statistical significance was considered through unpaired *t*-test (*α* = 0.05), with *p*-values adjusted for multiple comparisons using false discovery rate (FDR) correction. Finally, a chemical similarity enrichment analysis (ChemRICH) [41] was applied to assess the modulation of DAMs during brownie reformulation and SP processing, grouping them by chemical class.

On the other hand, raw and digested datasets from reformulated brownies were integrated through a regularized canonical correlation analysis (rCCA), using the MixOmics (v6.3.0) package in R(v4.3.2) [42]. The rCCA results were presented using a shrinkage XY-space score plot and a correlation circle plot (cut-off = 0.7), which allowed raw and digested metabolites to be multidimensionally correlated within a single canonical space. Finally, the relationships between raw and digested metabolites were observed using an integrative correlation network. A Pearson’s correlation coefficient threshold (r = 0.78) was applied for data visualization, and only significant correlated metabolites (*p* < 0.05) were shown.

## 3. Results and Discussion

### 3.1. Untargeted Metabolomic Profiling of Sweet Potato By-Products

**Figure 2:**
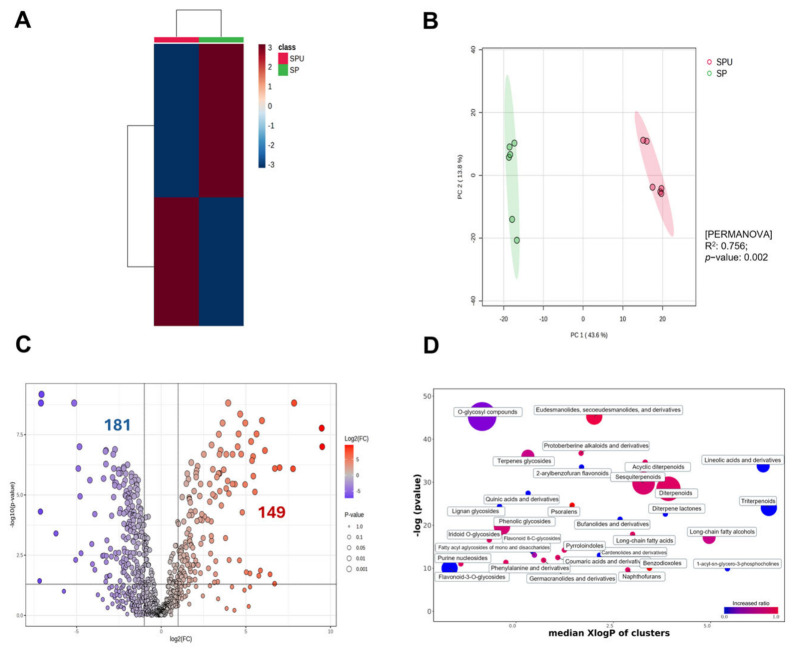
Untargeted metabolomic profiling of sweet potato by-products. (**A**) Hierarchical cluster analysis (HCA) of by-products. Fold-change values normalized to the mean abundance (Euclidean distances and Ward’s method) were considered. (**B**) Principal component analysis (PCA) of sweet potato by-products (R^2^ = 0.756, *p*-value = 0.002). Shaded areas represent the 95% confidence regions for each group. (**C**) Volcano analysis considering the untargeted profile of SPU in relation to SP. Dashed lines indicate the significance thresholds (*p* < 0.05 and FDR-adjusted *p*-value) (**D**) ChemRICH analysis of the DAMs derived from the volcano analysis ((log_2_FC) score ≥ |±2|; *p*-value < 0.05). The diameter of each node indicates the number of compounds represented for each corresponding chemical group, and the color of the node reflects the accumulation or degradation of the chemical class relative to SP. The *y*-axis represents the significance level (*p*-value), while the *x*-axis represents ascending polarity. The statistical relevance of each group was evaluated using the Kolmogorov–Smirnov test. Abbreviations: SP: sweet potato peel; SPU: sweet potato peel treated with UAE.

Untargeted metabolomics coupled with chemometrics was performed to decipher the overall chemical impact of UAE treatment on sweet potato peels. After annotation, a total of 1348 chemical features were reported across all raw samples (Table S1), and the main results of the analysis of by-products are summarized in Figure 2. An unsupervised hierarchical cluster analysis (HCA) was performed to investigate differences in normalized fold-change values of the two by-products (Figure 2A). This analysis, represented as an integrative heatmap, revealed a clear separation between the samples into two distinct clusters, indicating changes in the chemical composition of sweet potato peels. This metabolic modulation was further confirmed by the principal component analysis (PCA; Figure 2B), where the two by-products were separated based on UAE treatment. Furthermore, differences in the overall metabolomic profiles were statistically significant, as measured by PERMANOVA (R^2^ = 0.756; *p* = 0.002).

In order to study the behavior of UAE treatment on sweet potato peels, supervised modeling of the metabolic profile of the by-products was performed using volcano analysis (Figure 2C), obtaining as differentially accumulated metabolites (DAMs) the compounds that met the criteria of log_2_(FC) ≥ |±2| and *p* < 0.05. A total of 330 DAMs were reported after the application of UAE in the by-products (Table S2), of which 149 were accumulated and 181 depleted, indicating a mixed response in the modulation of metabolites induced by the extraction process. With the aim of identifying and examining the different chemical classes altered during treatment, DAMs were subjected to chemical enrichment analysis (ChemRICH) (Figure 2D; Table S3). The enrichment plot confirmed changes in the chemical profile of the by-products, showing an increase in terpenoid-related secondary metabolites, such as eudesmanolides, sesquiterpenoids, diterpenoids, and naphthopurans. Ultrasound-assisted extraction is capable of forming cavitation bubbles that collapse and induce fragmentation, cell disruption and pore formation in plant matrices [16]. This effect may enhance the release of certain terpenoid-like and lipophilic compounds that are normally captured in the pericarp tissues of sweet potato peel, the main reservoir of these compounds in the tuber [43]. This ability of UAE to recover terpenoids has been previously studied in different fruit peels such as citrus [44] and passion fruit [45].

On the other hand, ultrasonic extraction had a negative impact on the glycosylated compounds in sweet potato peel, including O-glycosylated metabolites, lignan glycosides, and flavonoid 8-C-and 3-O-glycosides. This reduction may be attributed to the chemical instability of glycosylated compounds under ultrasonic conditions, potentially leading to the deglycosylation of certain phenolic compounds. Cavitation bubbles during UAE treatment are capable of generating temperatures of up to 5000 K and pressures of 1000 atm [46], which may induce cleavage of O-glycosidic and C-glycosidic bonds under cavitation stress. Overall, these results highlight that UAE not only enhances the release of lipophilic and terpenoid-related compounds, but also induces structural modifications in more labile metabolites, ultimately reshaping the metabolomic profile of sweet potato by-products.

### 3.2. Nutritional Composition of Sweet Potato By-Products and Gluten-Free Brownies

The chemical composition of sweet potato peels and the reformulated GF brownies is presented in Table 2. Ultrasound-assisted extraction (UAE) significantly modified the nutritional profile of sweet potato peels. A marked increase in carbohydrates was observed, which can be attributed to the breakdown of the plant cell wall induced by cavitation, improving solvent penetration and solubilization of intracellular sugars and low molecular weight polysaccharides. Similar increases in extractable carbohydrate fractions have been reported after UAE in fruit and vegetable by-products due to improved cell wall disruption and diffusion phenomena [47,48,49]. On the other hand, a reduction in the content of SDF, IDF, and ash was observed due to the treatment. This reduction may be mainly associated with the extraction strategy, since SPU was produced from the hydroalcoholic extract recovered after separation of the solid phase, the final lyophilized powder was enriched in soluble, low-molecular-weight metabolites, while most structural component, including insoluble fiber fractions and mineral-rich residues, remained in the discarded solid phase. This preferential recovery of water-soluble components during UAE inherently leads to lower SDF, IDF, and ash contents in SPU compared to non-extracted SP.

Incorporation of control sweet potato by-products into GF brownies led to significant increases in soluble and insoluble dietary fiber, particularly in BSP16 formulation. These findings may be attributed to the high amount of fiber reported in the by-products. Other studies have shown that adding peels from other vegetables can enhance the fiber content in bakery products [50,51,52], improving their functional properties. In contrast, protein, fat and ash, contents did not show significant differences between formulations, indicating that the addition of up to 16% SP or SPU does not substantially affect the overall nutritional quality of the product.

Moreover, the reformulation of brownies with by-products exhibited no significant impact on the physicochemical properties of the brownies (Table S4), with only minor changes in color (ΔE) observed, particularly in the 16% formulations. These variations in L*, a*, and b* values are likely attributable to the natural pigmentation in carotenoids and anthocyanins of the sweet potato peel [53]. Finally, both pH and total titratable acidity (TTA) remained stable across all formulations, confirming that SP and SPU addition did not significantly alter the acid–base characteristics of the matrix.

### 3.3. Untargeted Metabolomic Profiling of Raw Gluten-Free Brownies

**Figure 3:**
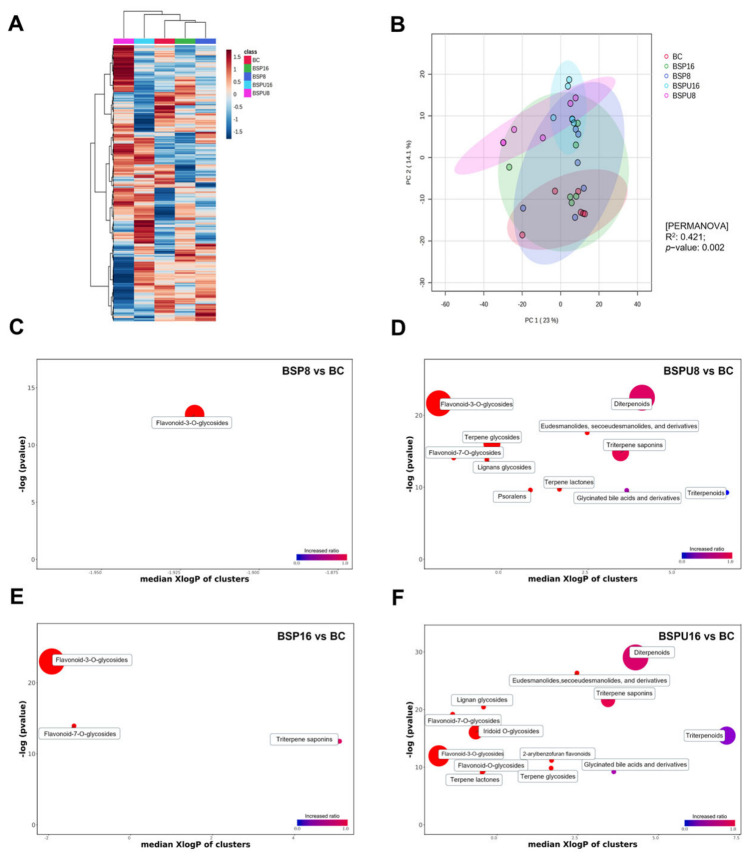
Untargeted metabolomic profiling of GF brownies. (**A**) Hierarchical cluster analysis (HCA) considering fold-change values normalized to the mean abundance (Euclidean distances; Ward’s method). (**B**) Principal component analysis (PCA) of GF brownies (R^2^ = 0.421, *p*-value = 0.002). Shaded areas represent the 95% confidence regions for each group. (**C**–**F**). ChemRICH analysis of the DAMs derived from the volcano analysis ((log_2_FC) score ≥ |±2|; *p*-value < 0.05) for BSP8 (**C**), BSPU8 (**D**), BSP16 (**E**), BSPU16 (**F**) in relation to BC. The diameter of each node indicates the number of compounds represented for each corresponding chemical group, and the color of the node reflects the accumulation or degradation of the chemical class relative to BC. The *y*-axis represents the significance level (*p*-value), while the *x*-axis represents ascending polarity. The statistical relevance of each group was evaluated using the Kolmogorov–Smirnov test. Abbreviations: BC: control brownie; BSP8: brownie with 8% SP; BSPU8: brownie with 8% SPU; BSP16: brownie with 16% SP; BSPU16: brownie with 16% SPU.

In order to understand the impact of reformulation with by-products on GF brownies, an untargeted metabolomic analysis coupled with multivariate analysis was performed (Figure 3). Unsupervised hierarchical cluster analysis (HCA) showed differences in the normalized fold-change values of the brownies (Figure 3A). This analysis revealed BSPU8 metabolome as the most distinctive from the rest of the brownies, clustering separately. In addition, the BSP8 and BSP16 formulations adopted similar chemical profiles in this analysis, indicating a possible role of UAE treatment in the differentiation of the samples. These metabolic changes were confirmed through principal component analysis (PCA; Figure 3B), which revealed a similar metabolome among all samples. However, marked differences were observed between the BSPU8 and BSPU16 formulations and the control brownie, confirming a key role of UAE treatment in the modulation of the chemical profile. To further conduct a more in-depth study of the reformulation with by-products, a supervised analysis was performed using Volcano analysis (log_2_(FC) ≥ |±2|; *p* < 0.05) (Figure S1; Table S5), followed by a chemical enrichment analysis (ChemRICH) (Figure 3C–F; Table S6) of the resulting differentially accumulated metabolites (DAMs) in relation to BC.

The incorporation of 8% sweet potato by-products led to the accumulation of a total of 20 DAMs, indicating an enrichment of the brownies due to the reformulation. In addition, ChemRICH analysis revealed a significant accumulation of flavonoid-3-O-glycosides (Figure 3C). Within this group, compounds such as quercetin 3-gentiobioside (log_2_(FC) = 3.1) and cameliaside B (log_2_(FC) = 2.2) showed pronounced increases, highlighting the effect of SP to the enhancement in flavonoid content during fortification. On the other hand, BSP16 formulation revealed the accumulation of 39 DAMs, indicating a dose–response effect in the fortification of brownies with SP. The lower number of DAMs in BSP8 compared to BSP16 could be attributed to lower metabolite abundance at reduced incorporation levels, resulting in decreased feature intensity and fewer metabolites surpassing detection and statistical thresholds rather than an absence of compounds. Chemical enrichment analysis also indicated the accumulation of flavonoid-3-O-glycosides after the addition of by-products; however, the appearance of flavonoid-7-O-glycosides and triterpene saponins was also detected (Figure 3E). These results indicate that fortification with SP has a positive effect on the flavonoid profile of the reformulated brownies. Several studies have shown that the sweet potato peel is a major source of flavonoids, especially myricetrins, quercetins, and apigenins [54,55]. It should be noted that the detected flavonoids found were predominantly present in glycosylated forms, reflecting their natural presence in plant tissues [56]. This suggests that the processing and baking of the brownies did not markedly affect the transformation or degradation of flavonoids present in SP.

Regarding brownies reformulated with UAE-treated by-products, BSPU8 showed a clear metabolic impact after reformulation, revealing up to 111 DAMs after volcano analysis. Specifically, the incorporation of 8% SPU caused a significant increase in flavonoid-O-glycosides and lignan glycosides, possibly due to the polyphenols present in sweet potato peel. In addition, an accumulation of diterpenoids, terpene glycosides, and eudesmanolides was detected, chemical classes that had been previously identified in the untargeted metabolomic profile of the by-products (Figure 2D). Similar results were reported in BSPU16 formulation, which showed an enrichment of diterpenoids, terpenoid glycosides, and flavonoid O-glycosides. These results indicate that UAE treatment was the main driver of metabolic changes in the reformulated brownies. This was confirmed in the Venn analysis (Figure S2), where up to 26 specific compounds accumulated in both UAE-treated formulations were identified, compared to 17 compounds associated with the addition of sweet potato peel regardless of treatment.

In general, these results indicate that treatment with UAE not only improved the extractability of specific chemical classes, as previously observed in the by-product analysis, but also promoted their effective incorporation into the GF brownie matrix, thereby increasing its overall chemical complexity. Moreover, the persistence of sweet potato-derived bioactive compounds present after baking suggest an adequate thermal stability within the food matrix, which is critical for their functional relevance. These findings are consistent with previous studies, reporting that the introduction plant by-products such as cocoa pods [57], artichoke stems [58] or red onion peels [59] into bakery products may contribute to an enrichment of the bioactive profile, enhancing the functional value of the final product.

### 3.4. Metabolomic Modulation and Antioxidant Activity of Reformulated Brownies After In Vitro Digestion

**Figure 4:**
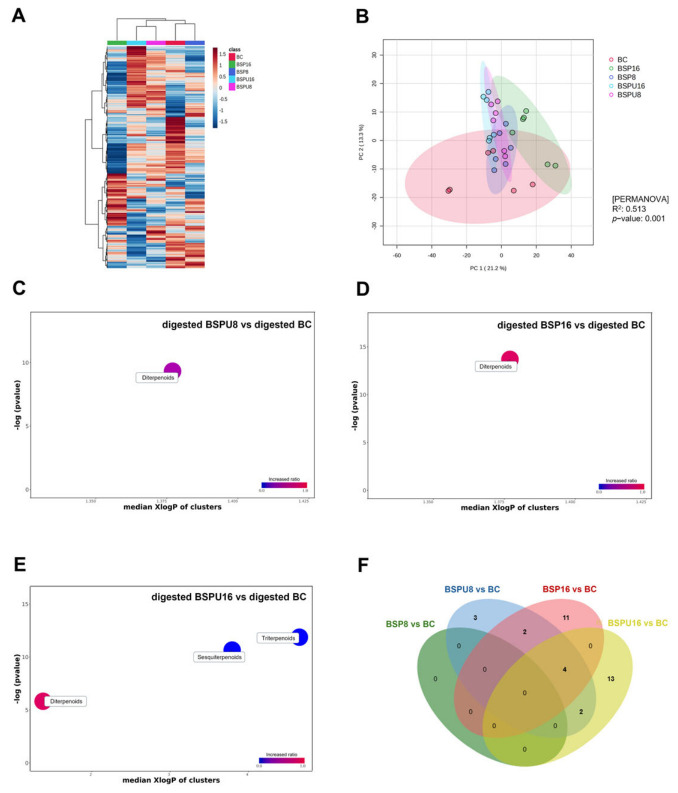
Untargeted metabolomic profiling of GF brownies after in vitro digestion. (**A**) Hierarchical cluster analysis (HCA) considering fold-change values normalized to the mean abundance (Euclidean distances; Ward’s method). (**B**) Principal component analysis (PCA) of digested GF brownies (R^2^ = 0.513, *p*-value = 0.001). Shaded areas represent the 95% confidence regions for each group. (**C**–**E**). ChemRICH analysis of the DAMs derived from the volcano analysis ((log_2_FC) score ≥ |±2|; *p*-value < 0.05) for BSPU8 (**C**), BSP16 (**D**), BSPU16 (**E**) in relation to digested BC. The diameter of each node indicates the number of compounds represented for each corresponding chemical group, and the color of the node reflects the accumulation or degradation of the chemical class relative to digested BC. The *y*-axis represents the significance level (*p*-value), while the *x*-axis represents ascending polarity. The statistical relevance of each group was evaluated using the Kolmogorov–Smirnov test. (**F**) Venn diagram analysis considering the accumulated DAMs of reformulated brownies in relation to digested BC. Abbreviations: BC: control brownie; BSP8: brownie with 8% SP; BSPU8: brownie with 8% SPU; BSP16: brownie with 16% SP; BSPU16: brownie with 16% SPU.

An untargeted metabolomic analysis was performed with the aim of exploring the reformulation of the brownies after an in vitro digestion following the INFOGEST 2.0 model. The unsupervised hierarchical cluster analysis (HCA; Figure 4A) showed differences in the fold-change values of the digested brownies, with the BSP16 metabolome differing from the other digested samples. In addition, the BSP8 formulation adopted a metabolomic profile similar to the control, suggesting that the percentage of the by-product incorporated into the brownies played an important role during digestion, in contrast to the raw samples where UAE treatment was more influential. Similar results were described in the PCA (Figure 4B), which indicated notable differences between the BSP16 formulations and the control brownie, confirming possible chemical modulations after the incorporation of by-products. However, a similar metabolome was observed among all formulations, possibly indicating that the digestive process reduced the differences between brownies. Subsequently, a supervised analysis was performed using Volcano analysis (log_2_(FC) ≥ |±2|; *p* < 0.05) (Figure S3; Table S7), followed by a ChemRICH analysis of the resulting DAMs (Figure 4C–E; Table S8), with the aim of studying the changes in depth of each formulation in relation to digested BC.

The incorporation of 8% SP led to minor changes in the metabolomic profile, showing the identification of one discriminant metabolite (Table S7). However, the formulation with UAE-treated SP induced a larger chemical modulation, revealing up to 31 metabolites. Among the accumulated metabolites, compounds such as monoacylglycerides or hydroxycinnamic acid derivatives were identified, whose accumulation may be attributed to cell disruption induced by UAE. On the other hand, the fortification of GF brownies with 16% SP exhibited greater impact on the metabolome, identifying up to 51 DAMs. Among the accumulated compounds, triterpenes and diterpenes stand out, as well as flavonoid derivatives. These compounds were detected in the raw samples, indicating that increasing the incorporation of SP to 16% enhanced the stability of some bioactive compounds after digestion. In particular, diterpenes and triterpenes have been shown to be resistant to the digestive environment and high acidity, exhibiting high bioaccessibility rates [60]. A similar impact regarding modulated DAMs was described for BSPU16, suggesting that the dose effect played a more important role than UAE treatment of the by-products. Notably, the Venn analysis (Figure 4F) showed that BSP16 and BSPU16 contained more exclusive metabolites than the other formulations, with 11 and 13 compounds accumulated uniquely, respectively. The BSPU16 formulation showed an accumulation of diterpenoids, as observed in the by-product-containing formulations. However, a reduction in sesquiterpenes and triterpenes was revealed, which is consistent with the described modulation of UAE in sweet potato by-products. This effect can be attributed to the cavitation effect, which can induce the degradation of terpenoids under certain power and time conditions, thereby reducing the recovery of these compounds [61]. The reduction in discriminant metabolites for BSP8 after digestion (from 20 in the raw state to 1 in the digested state) suggests that at 8% incorporation, the chemical contribution of SP was largely attenuated during gastrointestinal processing, whereas higher incorporation levels (16%) and UAE treatment provided greater chemical resilience. Overall, the differences between the digested brownies were smaller than in the raw samples, suggesting that during digestion, certain types of metabolites underwent partial transformation or degradation, although resistant compounds such as diterpenes, triterpenes, and certain flavonoids remained stable [62].

Furthermore, the impact on polyphenols after in vitro digestion in GF brownies was explored through semiquantitative analysis and total phenolic content (TPC) analysis, as well as their antioxidant effect in FRAP, ABTS, and DPPH assays (Table 3). The addition of sweet potato by-products exhibited a significant impact on flavonoid content after digestion, with the control formulation showing the lowest levels. In addition, a significant dose-dependent effect was mainly observed for this subfamily, with formulation BSP16 being the most enriched after digestion and showing the highest bioaccessibility (45.75%). These results on flavonoid digestibility are consistent with those reported by other authors, representing values of ~50% depending on chemical structure and glycosylation [63]. This effect observed in flavonoids may be attributed to the higher fiber content at the 16% incorporation level, as some studies suggest that dietary fiber can protect phenolic compounds during gastrointestinal digestion and promote their gradual release from the food matrix [64], thereby enhancing their bioaccessibility. In contrast, phenolic acids did not show significant differences between the digested formulations, despite having apparent bioaccessibility values close to or above 100%, due to their higher water solubility and lower molecular weight [65]. Apparent bioaccessibility values above 100% have been previously reported in the literature and may result from the release of bound phenolic compounds from the food matrix during digestion, increasing their detectable concentration in the bioaccessible fraction [66]. LMW phenolics were the abundant subfamily after digestion, representing a content of 4642–6555 mg 100 g^−1^ DW. In addition, the reformulation with by-products led to marked changes in this subclass, especially in the UAE-treated formulations, where there was a significant increase in digestibility (*p* < 0.05). This effect was also observed in the TPC values, where BSPU8 and BSPU16 formulations exhibited the highest content. UAE promotes acoustic cavitation, which disrupts plant cell walls and weakens interactions between phenolics and structural polysaccharides, enhancing their extractability and subsequent bioaccessibility. This mechanism has been widely described as a key factor explaining the improved recovery of phenolic compounds when ultrasound is applied [67,68]. Therefore, the higher digestibility of LMW phenolics and the increased TPC observed in BSPU8 and BSPU16 after digestion may indicate that UAE facilitated the liberation of smaller, more soluble phenolic compounds, making them more bioaccessible and less susceptible to losses during gastrointestinal digestion. These results were also supported by the TPC of the by-products, where the application of UAE revealed a significant increase (Table S9). Interestingly, increasing the incorporation level from 8% to 16% did not always result in a proportional increase in antioxidant capacity. For example, FRAP values in raw brownies increased from 900.28 to 1041.87 µmol TE 100 g^−1^ FW in BSPU8 and BSPU16, respectively, whereas DPPH values decreased from 729.21 to 682.36 µmol TE 100 g^−1^ FW. This suggests that the relationship between sweet potato peel concentration and antioxidant activity is not strictly linear. A possible explanation is the higher dietary fiber content provided by the 16% incorporation level, which may retain or entrap part of the phenolic compounds within the food matrix, limiting their extractability and interaction with the radicals used in antioxidant assays despite their presence in the product [69]. Finally, the increase in phenolic compounds translated into significant improvements in antioxidant capacity in both raw and digested brownies. FRAP results showed that BSPU16 exhibited the highest antioxidant capacity in both raw and digested samples, suggesting a combined effect of dose (16% incorporation) and UAE treatment. The improvement in antioxidant activity can be mainly attributed to the higher polyphenol content resulting from sweet potato peel incorporation. Low-molecular-weight phenolics are known to exhibit strong antioxidant activity due to their high redox potential and greater solubility [70]. Moreover, sweet potato peel has been widely reported as a rich source of bioactive phenolics with remarkable antioxidant capacity, particularly hydroxycinnamic acid derivatives and flavonoids, contributing significantly to reducing power and radical scavenging activity [71].

### 3.5. Computational Integration of Raw and Digested Metabolomic Datasets

**Figure 5:**
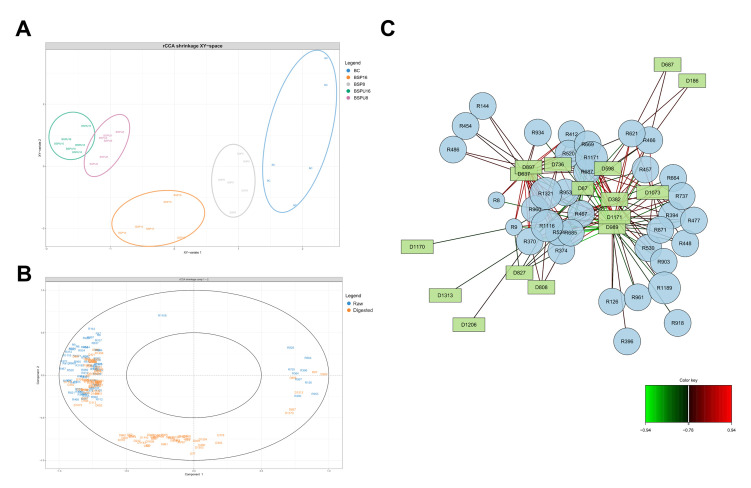
Regularized canonical correlation analysis (rCCA) on the integration of raw and digested datasets of GF brownies. (**A**) Shrinkage XY-space score plot derived from rCCA integration of raw and digested brownies. (**B**) Correlation circle plot derived from rCCA raw and digested metabolomes (cut-off: 0.7). The complete list of metabolites and their respective codes is detailed in Table S10. (**C**) Integrative network on the significant correlations between raw and digested metabolites (cut-off = 0.78; *p* < 0.05). Node shape indicates the metabolite type (squares: raw metabolites; circles: digested metabolites), while edge color represents the correlation value according to the color scale shown. Correlation values are displayed in Table S11. Abbreviations: BC: control brownie; BSP8: brownie with 8% SP; BSPU8: brownie with 8% SPU; BSP16: brownie with 16% SP; BSPU16: brownie with 16% SPU.

A multivariate integrative analysis using regularized canonical correlation analysis (rCCA; Figure 5) was conducted to explore the relationships between the metabolomes of raw and in vitro digested GF brownies. This approach provides a global view of how the inclusion of sweet potato by-products and the application of ultrasound-assisted extraction (UAE) influenced metabolite profiles during in vitro digestion. The resulting score plot revealed five clearly separated clusters, highlighting pronounced differences among the formulations (Figure 5A). UAE treatment appeared to enhance the differentiation along the first canonical axis, indicating that this processing method amplified the metabolic divergence of the samples. Moreover, the proportion of sweet potato added contributed to the separation, with both untreated and UAE-treated 16% formulations showing the greatest distinction from the control. In addition, the control brownies displayed a wider spread within the plot, indicating differences between their raw and digested metabolomes. In contrast, reformulated brownies exhibited a tighter clustering of raw and digested samples, suggesting that the addition of sweet potato by-products, particularly when combined with UAE, mitigated metabolic alterations during digestion and favored the retention of bioactive compounds. These observations indicate that formulation and processing not only drive compositional differences but also contribute to the preservation of metabolites under gastrointestinal conditions. A correlation circle plot derived from rCCA model (Figure 5B) provided deeper insight into the variables contributing to the overall covariance between raw and digested metabolites. The comprehensive list of metabolites and their respective codes are detailed in Table S10. This analysis revealed a clear distribution of raw metabolites on the negative side of the *y*-axis, while digested metabolites exhibited a broader distribution along the *x*-axis. Digested metabolites that did not correlate with the raw fraction of the brownies were mainly associated with conjugated bile acids, mono- and triacylglycerides, and small peptides. These compounds are either formed during digestion through enzymatic activity or the action of bile salts or are highly labile under gastrointestinal conditions, being susceptible to hydrolysis, oxidation, or enzymatic transformation. Consequently, their abundance patterns do not align with those of the raw metabolites. In contrast, digested metabolites that showed positive correlations with raw metabolites were predominantly complex secondary metabolites such as triterpenoids, saponins, and steroidal derivatives. These compounds possess rigid polycyclic structures that are relatively resistant to acidic and enzymatic conditions, preserving their core structure during digestion and maintaining a high bioaccessibility [72].

These results were confirmed by the integrative network (Figure 5C; Table S11), which showed a structured organization centered on a few highly connected digested hubs. In particular, diterpenoid-related metabolites such as 20-deoxyingenol (D382), 7-beta-hydroxylathyrol (D598), and perivine (D1171) acted as central nodes, correlating broadly with raw terpenoid families and certain flavonoids. These findings align with the metabolomic profile of raw and digested samples. Although sweet potato incorporation modulated glycosylated flavonoids in raw brownies, many of these phenolic compounds are susceptible to deglycosylation and oxidation during digestion, reducing their discriminative role post-digestion [73]. As a result, differences among formulations after digestion were mainly driven by a relatively higher abundance of terpenoid-related metabolites, reflecting their greater chemical stability and lipophilic character [74]. Importantly, the prominence of diterpenoid hubs in the rCCA network is coherent with the previously observed increase in triterpenoids after UAE treatment of sweet potato by-products. These results indicate that UAE not only improves the initial extraction of bioactive compounds, but also contributes to their structural persistence and bioaccessibility during gastrointestinal digestion. On the other hand, glycerol myristate (D897) also emerged as a key derived node, showing strong positive correlations with terpenoids and phenolic derivatives from raw brownies. These correlations may indicate the possible hydrolysis of lipids during digestion and the subsequent formation of micelles, which facilitates the co-solubilization of hydrophobic secondary metabolites [75].

### 3.6. Biological Activities of Gluten-Free Brownies After In Vitro Digestion

**Figure 6:**
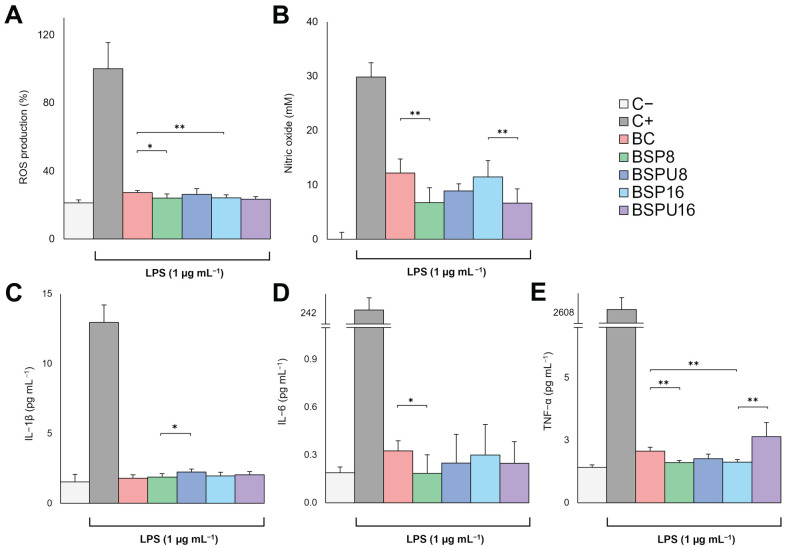
Biological activities of digested GF brownies in a RAW264.7 cell line under an LPS-induced inflammatory model. Reactive oxygen species (ROS) generation (**A**), nitric oxide (**B**), and pro-inflammatory cytokine production, including IL-1β (**C**), IL-6 (**D**), and TNF-α (**E**), following treatment with digested brownie samples. Data are presented as mean ± SD. Differences were assessed among independent sample groups using Student’s *t*-test. Significance levels are indicated as follows: *: *p* < 0.05; **: *p* < 0.01. Abbreviations: BC: control brownie; BSP8: brownie with 8% SP; BSP16: brownie with 16% SP; BSPU8: brownie with 8% SPU; BSPU16: brownie with 16% SPU; C−: negative control; C+: positive control; IL: interleukin; LPS: lipopolysaccharide; ROS: reactive oxygen species; TNF: tumor necrosis factor.

The antioxidant and anti-inflammatory properties of digested brownies were evaluated using a cell culture with RAW264.7 macrophages after the induction of inflammation by LPS (Figure 6). The production of reactive oxygen species (ROS) was measured in the cellular model following the application of the digests, with the results presented relative to an LPS-induced positive control (Figure 6A). The inclusion of digested brownies led to a significant reduction in ROS production compared to the positive control, indicating a protective effect against oxidation. Furthermore, the incorporation of sweet potato peel at both 8% and 16% resulted in significant changes compared to BC. These findings are consistent with the TPC and FRAP results observed after digestion, where the formulations containing sweet potato obtained higher values (Table 3). Higher FRAP values are associated with a greater capacity to donate electrons, which may protect cells from oxidative stress and help neutralize intracellular ROS [76]. Furthermore, it has been demonstrated that the high polyphenol content of SP-enriched formulations, particularly that of flavonoids may activate the Keap1/Nrf2/NQO1 pathway in RAW264.7 macrophages, preventing the oxidative damage caused by LPS [77].

In addition, the release of inflammatory-related compounds such as nitric oxide (NO) and pro-inflammatory cytokines such as interleukin (IL)-1β, IL-6, and tumor necrosis factor (TNF)-α by macrophages was also determined. The addition of 8% sweet potato peel had a direct effect on reducing the release of NO, IL-6, and TNF-α compared to the control (Figure 6B–E), suggesting a potential anti-inflammatory effect due to the polyphenolic compounds present in the by-products after digestion. Recent studies have reported that phenolic compounds present in foods can induce an anti-inflammatory response in RAW264.7 macrophages through the inhibition of NF-κB signaling and subsequent reduction of pro-inflammatory cytokines [78,79]. However, higher levels of SP incorporation did not produce a greater anti-inflammatory effect in the cellular model. These findings are consistent with the results regarding TPC and antioxidant activity, suggesting a possible interaction between higher dietary fiber content and phenolic compounds that could limit the bioavailability of specific bioactive compounds in the cell culture [80]. Finally, the application of UAE to sweet potato by-products displayed a mixed effect on inflammation, as it increased the release of TNF-α and IL-1β while reducing NO. This heterogeneous anti-inflammatory response could be attributed to the metabolomic shifts observed following the digestion of reformulated brownies, where a reduction in triterpenes and diterpenes was observed, particularly in BSPU16 (Figure 4E). These classes of terpenoids have been widely associated with anti-inflammatory activity, including the inhibition of pro-inflammatory mediators [81]. Therefore, their decrease following treatment with UAE could partly explain the attenuated or differential cytokine response observed.

### 3.7. Sensory Evaluation of Gluten-Free Brownies

**Figure 7:**
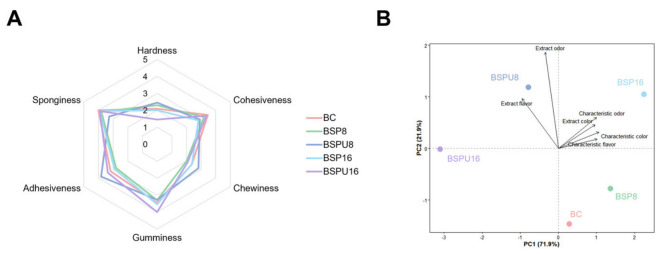
Sensory analysis of GF brownie formulations. (**A**) Radar plot showing the texture-related attributes for the different brownie samples. (**B**) Principal component analysis (PCA) biplot representing the distribution of brownie samples according to their extract-related profiles and the contribution of each attribute to sample differentiation. Abbreviations: BC: control brownie; BSP8: brownie with 8% SP; BSPU8: brownie with 8% SPU; BSP16: brownie with 16% SP; BSPU16: brownie with 16% SPU.

The results of the sensory evaluation of the GF brownies evaluated by the trained panelists are presented in Figure 7 [82,83]. Regarding texture-related attributes, none of the formulations showed significant changes in hardness, cohesiveness, adhesiveness, gumminess, sponginess, or chewiness (Figure 7A, Table S12), indicating a minimal impact after the addition of sweet potato peels.

Regarding the PCA of sensory attributes related to characteristic and extract-associated perceptions (Figure 7B), BSP8 clustered closest to the control brownie (BC), indicating that the incorporation of 8% sweet potato peel caused minimal alterations in the extract-related sensory profile of the GF brownies. Similar findings have been previously reported after the incorporation of other agri-food by-products into bakery products, where moderate enrichment levels preserved the typical sensory profile and maintained good acceptability, as observed with apple [84] and coffee [85] by-products. In addition, BSP16 appeared slightly separated from the control, although it remained aligned with characteristic flavor, color, and odor attributes, suggesting a good sensory acceptance despite the higher incorporation level. In contrast, formulations containing UAE-treated by-products showed a greater association with extract-related sensory descriptors, particularly BSPU16, which was more strongly aligned with extract flavor. These findings indicate that UAE treatment exerted a certain influence on sensory perception, probably due to the enhanced release of volatile and aromatic compounds promoted by cavitation-induced cell disruption during ultrasound processing. It has been previously described that UAE can intensify flavor notes through the improved liberation of aroma-active compounds and secondary metabolites from plant tissues [86], which may explain the slightly more pronounced extract-associated sensory profile observed in UAE-treated formulations.

## 4. Conclusions

The use of sweet potato by-products in gluten-free brownie reformulation demonstrated strong potential for developing nutritionally enhanced and functionally relevant bakery products. This approach enriched the matrix with bioactive compounds, particularly flavonoid glycosides that remained stable after processing and digestion. These compositional improvements were associated with reduced oxidative stress and modulation of inflammatory responses in a RAW264.7 model. The 8% incorporation level provided a balanced condition, ensuring functional delivery without compromising bioavailability. Sensory analysis confirmed that sweet potato peel incorporation did not negatively affect texture or overall acceptability, maintaining a profile comparable to the control and preserving extract-related perception. However, it should be noted that sensory evaluation was based on a trained panel descriptive analysis and did not include hedonic testing; therefore, consumer acceptability and preference cannot be directly inferred from the present results.

In parallel, ultrasound-assisted extraction showed both advantages and limitations. Although the SPU fraction obtained after UAE exhibited a lower total fiber content, reflecting its enrichment in extractable soluble components, it enhanced the recovery and bioaccessibility of low molecular weight phenolics, indicating a modulatory effect on the bioactive profile. From a sensory perspective, UAE slightly increased extract-associated perception due to enhanced release of aroma compounds, while overall acceptability remained stable. However, structural modifications in metabolites may explain the more heterogeneous biological responses observed, highlighting the need for an integrated evaluation of the process beyond extraction yield.

Overall, these results supported the valorization of sweet potato peel by-products combined with emerging technologies such as ultrasound-assisted extraction as a promising pathway for the reformulation of bakery products into next-generation functional foods. Furthermore, the application of metabolomics proved to be a key tool for understanding how bioactive compounds were transformed during processing and digestion, enabling a more rational and targeted design of foods with demonstrated health related benefits. It should be noted that the sweet potato peels used in this study were obtained by manual peeling, which may not be fully representative of industrially generated peel by-products in terms of peeling depth, flesh-to-peel ratio, and compositional consistency. Future studies should evaluate peels obtained under controlled industrial conditions to strengthen the translational relevance of the findings.

## Figures and Tables

**Table 1:** Formulation of GF brownies (g).

Ingredients	BC	BSP8	BSPU8	BSP16	BSPU16
Sugar	175	175	175	175	175
Whole milk	110	110	110	110	110
EVOO	83	83	83	83	83
Buckwheat flour	70	70	70	70	70
Cocoa powder 70%	75	75	75	75	75
Chocolate chips	75	75	75	75	75
Egg	60	60	60	60	60
SP		5.6		11.2	
SPU			5.6		11.2
Total	648	653.6	653.6	659.2	659.2

The percentage of SP and SPU addition was calculated relative to the weight of buckwheat flour used in the formulation. Abbreviations: BC: control brownie; BSP8: brownie with 8% SP; BSPU8: brownie with 8% SPU; BSP16: brownie with 16% SP; BSPU16: brownie with 16% SPU; SP: sweet potato peel; SPU: sweet potato peel treated with UAE.

**Table 2:** Proximate composition of brownies and sweet potato by-products.

	Gluten-Free Brownies					Sweet Potato by-Products	
	BC	BSP8	BSPU8	BSP16	BSPU16	SP	SPU
Energy content (kcal 100 g^−1^ FW)	381.16 ± 5.00 ab	376.06 ± 5.73 ab	390.25 ± 4.54 a	369.47 ± 5.9 b	384.24 ± 8.64 a	297.07 ± 2.54 b	355.18 ± 3.85 a
Moisture (g 100 g^−1^ FW)	21.96 ± 0.64 a	22.66 ± 1.02 a	20.82 ± 0.87 a	22.94 ± 0.60 a	22.14 ± 0.91 a	8.26 ± 0.34 a	6.10 ± 0.68 b
Ash (g 100 g^−1^ FW)	1.63 ± 0.06 a	1.69 ± 0.07 a	1.70 ± 0.08 a	1.73 ± 0.11 a	1.77 ± 0.04 a	7.08 ± 0.21 a	5.08 ± 0.35 b
Fat (g 100 g^−1^ FW)	17.54 ± 0.55 a	17.45 ± 0.48 a	18.28 ± 0.28 a	16.74 ± 0.83 a	18.07 ± 0.84 a	1.78 ± 0.11 a	0.20 ± 0.04 b
Protein (g 100 g^−1^ FW)	5.88 ± 0.11 a	5.85 ± 0.23 a	5.77 ± 0.04 a	5.94 ± 0.23 a	5.79 ± 0.29 a	5.56 ± 0.01 a	5.12 ± 0.01 b
Carbohydrates (g 100 g^−1^ FW)	48.81 ± 0.27 ab	45.39 ± 0.94 ab	47.79 ± 0.74 a	44.80 ± 0.56 b	46.90 ± 1.57 ab	51.95 ± 0.87 b	82.87 ± 0.90 a
SDF (g 100 g^−1^ FW)	1.50 ± 0.02 b	2.06 ± 0.07 ab	1.53 ± 0.20 b	2.60 ± 0.26 a	1.43 ± 0.17 b	7.83 ± 0.21 a	0.64 ± 0.25 b
IDF (g 100 g^−1^ FW)	4.67 ± 0.18 ab	4.91 ± 0.55 ab	4.10 ± 0.10 b	5.26 ± 0.04 a	3.90 ± 0.23 b	17.54 ± 0.53 a	0.00 ± 0.00 b
TDF (g 100 g^−1^ FW)	6.18 ± 0.19 bc	6.97 ± 0.48 ab	5.63 ± 0.30 bc	7.86 ± 0.22 a	5.32 ± 0.41 c	25.37 ± 0.74 a	0.64 ± 0.25 b

Results were reported as mean ± standard deviation. Different letters within the same row indicate significant differences between brownie formulations based on Tukey’s HSD test (*p* < 0.05), while differences among sweet potato by-products were assessed using Student’s *t*-test (*p* < 0.05). Abbreviations: BC: control brownie; BSP8: brownie with 8% SP; BSPU8: brownie with 8% SPU; BSP16: brownie with 16% SP; BSPU16: brownie with 16% SPU; FW: fresh weight; IDF: insoluble dietary fiber; SDF: soluble dietary fiber; SP: sweet potato peel; SPU: sweet potato peel treated with UAE; TDF: total dietary fiber.

**Table 3:** Semi-quantification of phenolic compounds and antioxidant activities of raw and digested GF brownies.

	Levels	BC	BSP8	BSPU8	BSP16	BSPU16
Flavonoids (mg LE 100 g^−1^ DW)	Raw	124.88 ± 25.88 Aa	119.75 ± 32.48 Aa	135.53 ± 50.43 Aa	101.77 ± 7.51 Aa	150.85 ± 55.30 Aa
	Digested	37.33 ± 4.68 Cb	39.30 ± 3.85 BCb	41.15 ± 5.00 BCb	46.56 ± 4.21 ABb	49.93 ± 0.84 Ab
	BA (%)	29.89 ± 5.03 B	32.82 ± 3.22 B	30.36 ± 3.69 B	45.75 ± 4.14 A	33.10 ± 0.56 B
Phenolic acids (mg CE 100 g^−1^ DW)	Raw	200.02 ± 46.81 Aa	205.29 ± 53.45 Aa	214.42 ± 35.24 Aa	211.16 ± 75.43 Aa	259.95 ± 53.06 Aa
	Digested	175.96 ± 13.06 Ab	224.43 ± 49.26 Aa	233.34 ± 34.86 Aa	268.36 ± 93.42 Aa	262.41 ± 48.14 Aa
	BA (%)	87.97 ± 6.53 A	109.32 ± 23.99 A	108.82 ± 16.26 A	127.09 ± 44.24 A	100.94 ± 18.52 A
LMW phenolics and other polyphenols (mg SE 100 g^−1^ DW)	Raw	11,587.90 ± 2093.69 ABa	12,313.81 ± 1315.38 Aba	11,149.89 ± 1830.52 Ba	15,537.70 ± 1620.31 Aa	11,516.93 ± 4020.83 ABa
	Digested	4646.53 ± 1404.56 Bb	4718.62 ± 448.97 Bb	6554.94 ± 1338.20 Aa	6028.00 ± 526.55 ABb	6062.73 ± 204.55 ABb
	BA (%)	40.10 ± 12.12 BC	38.32 ± 3.65 C	58.79 ± 12.00 A	38.80 ± 3.39 C	52.50 ± 1.68 AB
TPC (mg GAE 100 g^−1^ FW)	Raw	272.24 ± 9.17 Aa	297.51 ± 20.89 Aa	335.46 ± 14.97 Aa	287.34 ± 33.79 Aa	288.52 ± 32.02 Ab
	Digested	286.27 ± 54.32 Ba	307.00 ± 8.43 ABa	348.36 ± 30.16 ABa	333.53 ± 18.13 ABa	401.97 ± 51.44 Aa
FRAP (µmol TE 100 g^−1^ FW)	Raw	760.96 ± 52.57 Ba	878.36 ± 81.68 ABa	900.28 ± 18.59 ABa	963.08 ± 120.88 ABa	1041.87 ± 73.94 Aa
	Digested	590.54 ± 48.21 Ca	710.13 ± 10.27 Bb	744.00 ± 48.46 ABb	787.51 ± 15.64 ABa	804.00 ± 22.96 Ab
DPPH (µmol TE 100 g^−1^ FW)	Raw	543.51 ± 80.45 Aa	644.81 ± 87.67 Aa	729.21 ± 132.40 Aa	634.85 ± 123.76 Aa	682.36 ± 118.67 Aa
	Digested	500.66 ± 21.88 Aa	490.58 ± 60.90 Aa	591.74 ± 52.11 Aa	564.16 ± 6.50 Aa	531.48 ± 26.12 Aa
ABTS (µmol TE 100 g^−1^ FW)	Raw	824.07 ± 63.55 Ba	971.88 ± 23.30 Aa	955.40 ± 19.46 Aa	955.65 ± 12.13 Aa	946.28 ± 18.86 Aa
	Digested	571.35 ± 38.47 Ab	640.48 ± 30.57 Ab	617.10 ± 50.25 Ab	649.00 ± 38.95 Ab	599.03 ± 29.07 Ab

Results were expressed as the mean ± standard deviation. A–C: different letters within the same row indicate significant differences between brownie formulations (Tukey HSD; *p* < 0.05); a–b: different letters within the same column indicate significant differences between raw and digested brownies (Student’s *t*-test; *p* < 0.05). Abbreviations: ABTS: 2,2′-Azino-bis(3-ethylbenzothiazoline-6-sulfonic acid); BA, percentage of bioaccessibility after in vitro gastrointestinal digestion; BC: control brownie; BSP8: brownie with 8% SP; BSPU8: brownie with 8% SPU; BSP16: brownie with 16% SP; BSPU16: brownie with 16% SPU; CE: chlorogenic acid equivalents; DPPH: 2,2-Diphenyl-1-picrylhydrazyl; DW: dry weight; FRAP: ferric reducing antioxidant power; FW: fresh weight; GAE: gallic acid equivalents; LE, luteolin equivalents; SE, sesamin equivalents; LMW: low-molecular-weight; TPC: total phenolic content; TE: Trolox equivalents.

## Data Availability

The original contributions presented in this study are included in the article/supplementary material. Further inquiries can be directed to the corresponding author(s).

## Supplementary Materials

The following supporting information can be downloaded at: https://www.mdpi.com/article/10.3390/antiox15060753/s1, Table S1: Dataset of putatively annotated compounds of raw and digested gluten-free brownies and sweet potato by-products after untargeted UHPLC-QTOF-HRMS analysis; Table S2: Discriminant accumulated metabolites (DAMs) (log_2_(FC) ≥ ± 2 and *p* < 0.05) of sweet potato by-products from volcano analysis in relation to SP; Table S3: ChemRICH results based on discriminant metabolites (log_2_(FC) ≥ ± 2 and *p *< 0.05) of sweet potato by-products; Table S4: Physicochemical characteristics of gluten-free brownies; Table S5: Discriminant accumulated metabolites (DAMs) (log_2_(FC) ≥ ± 2 and *p* < 0.05) of gluten-free brownies from volcano analysis in relation to BC; Table S6: ChemRICH results based on discriminant metabolites (log_2_(FC) ≥ ± 2 and *p* < 0.05) of gluten-free brownies; Table S7: Discriminant accumulated metabolites (DAMs) (log_2_(FC) ≥ ± 2 and *p* < 0.05) of digested gluten-free brownies from volcano analysis in relation to digested BC; Table S8: ChemRICH results based on discriminant metabolites (log_2_(FC) ≥ ± 2 and *p* < 0.05) of gluten-free brownies after in vitro digestion; Table S9: Semi-quantification of phenolic compounds and antioxidant activities of sweet potato by-products; Table S10: Metabolites derived from rCCA raw and digested metabolomes of gluten-free brownies (cut-off: 0.7); Table S11: Pearson correlation coefficients (cut-off = 0.78) on the rCCA-mediated integration of raw and digested metabolites of gluten-free brownies; Table S12: Sensory analysis of gluten-free brownies; Figure S1: Supervised volcano analysis for BSP8 (A), BSPU8 (B), BSP16 (C), and BSPU16 (D) in relation to BC. Thresholds of (log_2_FC) score ≥ ∣± 2∣ and *p*-value < 0.05 were applied; Figure S2: Venn diagrams considering discriminant accumulated (A) and depleted (B) metabolites (DAMs) of reformulated brownies in relation to BC. The identified DAMs were derived from the volcano plot analysis of the different formulations (Figure S1) based on the thresholds of (log_2_FC) score ≥ ∣± 2∣ and *p*-value < 0.05; Figure S3: Supervised volcano analysis for digested BSP8 (A), BSPU8 (B), BSP16 (C), and BSPU16 (D) in relation to digested BC. Thresholds of (log_2_FC) score ≥ ∣± 2∣ and *p*-value < 0.05 were applied.
